# Perceptions and Treatment of Precocious Puberty: A Questionnaire Survey among Caregivers in South Korea

**DOI:** 10.1155/2022/9413188

**Published:** 2022-07-21

**Authors:** Soo Bo Shim, Ji Hyun Song, Hye Lim Lee

**Affiliations:** Department of Korean Pediatrics, College of Korean Medicine, Daejeon University, 75 Daedeok-Daero, 176 Beon-Gil, Seo-Gu, Daejeon, Republic of Korea

## Abstract

This study aims to provide the basis for developing complementary and alternative treatment approaches for precocious puberty (PP) by examining caregivers' awareness, along with their preferences and expectations from various treatments. It included data collected between July 2019 and March 2020, from 175 caregivers of children who were undergoing or had undergone treatment for PP. The questionnaire comprised 53 questions on the awareness, acquisition of information, perceptions of treatments, and concerns about PP, as well as awareness of habit management (HM) and children's actual habits. The collected data were analysed using the Chi-square test, *t*-tests, and one-way analysis of variance. The caregivers responded that PP could be recognized on the basis of breast development (79.8%) in girls and hair and body odor (73.1%) in boys. However, 86.9% of respondents were getting inaccurate information from the Internet. With respect to PP treatment approaches, 16% of respondents hoped to manage PP through conventional treatment (CT), 21.1% preferred traditional Korean medicine (TKM), and 62.3% preferred HM. Expectations of the effectiveness were highest in HM, followed by CT and TKM. Only the TKM group had statistically significant higher expectations than the nontreatment experience group (*F* = 4.566, *p*=0.004). The caregivers were concerned about the side effects and high costs of CT. Around 43% of respondents had difficulties with the management of their children's diet, 64.6% with exercise, and 57.1% with smart device usage. Clinicians should be aware of caregivers' information acquisition patterns, preferences and expectations of various treatments, and their concerns. Considering these results, clinicians should try to establish more appropriate treatment plans for children with PP.

## 1. Introduction

Recent reports in the Republic of Korea revealed that children are maturing at an earlier age owing to several factors, such as westernized diets, increased exposure to environmental hormones, and stress [1]. Puberty is the most dramatic period of human development during which mental and social changes occur, along with rapid physical growth of the body [2]. Given that precocious puberty (PP) has been found to cause physical, mental, and social problems [3], there is an increasing interest in treating this condition. Consequently, social costs for PP are increasing, given that the prevalence of PP increased from 29,251 patients in 2010 to 108,576 in 2019 and the total insurance expenses increased from 25,716,431 won in 2010 to 77,028,458 won in 2019 [4].

PP is defined as the occurrence of secondary sexual characteristics (SSC), two standard deviations above average, usually with breast development prior to 8 years of age in girls and testicular development prior to 9 years of age in boys. PP is usually caused by the premature activation of the hypothalamic-pituitary-gonadal axis, which is described as central PP. Approximately 90–95% of girls and a minority of boys have an idiopathic aetiology and are classified as idiopathic central PP (ICPP) [5].

PP causes physical and psychological deficits in children. In addition, several studies have shown that girls with PP are more likely to experience depression, as well as social and psychological problems, such as early pregnancy, sexual abuse, alcohol or drug addiction, and behavioural disorders. Other studies have shown that PP increases the incidence of diabetes, cardiovascular diseases, and breast cancer [2, 6–8]. In addition, it causes advanced bone age and increased growth rate that result in premature fusion of the growth plates, which, in turn, leads to short final adult height [9].

Currently, the goals of treatment are to maintain SSC rates similar to that of peers, minimize the loss of final adult height, and reduce psychosocial problems [10]. The conventional treatment (CT) for ICPP is gonadotropin-releasing hormone agonist (GnRHa). However, complementary and alternative medicine (CAM) treatment is often considered despite the following concerns: reluctance to start artificial hormone therapy at an early age, fear of potential side effects, discomfort of getting an injection every four weeks, and poorer outcomes than expected with regards to the improvement of final adult height [11]. Traditional Korean medicine (TKM) has been used for the treatment of various diseases and is currently one of the most popular CAM [12] treatments. ICPP, which accounts for the highest percentage of PP and is free from other identifiable diseases, can benefit from TKM [11]. Several studies too have reported the effectiveness of TKM on ICPP [13–15].

The management of a child's environment or habits is largely influenced by caregivers, and treatment is also dependent on caregivers' choices [16]. In the prevention, management, and treatment choices of PP, the role of caregivers is significantly direct and important [17]. Therefore, considering caregivers' perceptions and attitudes regarding the disease has a significant impact on establishing strategies for treating PP.

Previous studies on PP have mainly focused on the effectiveness of GnRHa and herbal medicines [13, 14, 18, 19], psychological characteristics of patients (not their caregivers) [20], and causes of PP [1]. Most studies on the caregivers of patients with PP have focused on the stress level and quality of life among caregivers [21]. Moreover, only limited studies examined perceptions of the disease, treatment options, and the habits of those diagnosed with PP. There have been no reports of studies investigating caregivers' perceptions of PP or their preference for PP treatment (including CT, CAM such as TKM, and habit management (HM)). Therefore, this study was conducted to provide useful basic data for clinicians to establish more appropriate treatment approach strategies for children with PP by identifying caregivers' awareness, preference expectations, and concerns about various treatments.

## 2. Materials and Methods

### 2.1. Research Questions

The following five clinical questions were developed to obtain significant information and assess parental awareness:

1. How much do caregivers know about PP?
2. Where do caregivers obtain information regarding PP, and how much do they trust this information?
3. What is the level of caregivers' preference for CT/TKM/HM as a treatment alternative?

### 2.2. Development of the Questionnaire

We drafted a questionnaire based on the research questions and modified the questions by referring to related previous studies. To establish the questionnaire's validity as a tool, we obtained reviews from Traditional Korean Paediatricians as well as experts in medical statistics and research. The questionnaire included the following domains: general characteristics of respondents, awareness of PP, acquisition of information about PP, perception of treatment for PP (including preference as a treatment choice), concerns about PP, and awareness of HM and children's actual habits (the entire survey form has been included in Supplementary Materials). Because most of the questions comprised nominal scale ones, there was a problem with evaluating their reliability and validity. Therefore, both factor and reliability analyses were conducted on parts of the questionnaire items. In this study, Cronbach's *α* value was 0.840, Kaiser–Meyer–Olkin measure of sampling adequacy was 0.797, Bartlett's test of sphericity result was 538.628, and *p* < 0.001.

### 2.3. Survey Process

We conducted a survey on caregivers who had visited TKM institutions for PP treatment between July 2019 and March 2020 in Daejeon and Seoul, South Korea, and invited a total of 200 caregivers to participate. To arrive at the number of participants required for this study, we used the G-power version 3.1.9.7 program, included an effect size of 0.25, *α* level of 0.05, and power value of 0.8, and calculated the sample size as 159. However, considering the dropout rate and the convenience of the survey, the total number of participants was determined as 200.

To assess parental perceptions of PP and its treatment, we included caregivers whose children were undergoing or had undergone treatment for PP. We excluded questionnaires in which information on children's birth dates was missing, as well as those that did not appropriately answer the questions.

### 2.4. Statistical Analysis

All the responses were processed using the Statistical Package for the Social Sciences (SPSS) version 23.0 (IBM Corp. Armonk, NY, USA), and demographic characteristics were analysed by calculating frequencies, percentages, means, and standard deviations A Chi-square test was conducted to compare the treatment preferences according to the treatment experiences. The independent *t*-test and one-way analysis of variance were conducted to compare the stress levels and expectations of the effectiveness based on caregivers' treatment experiences. The results were recognized as significant if the *p* value was less than 0.05. Unanswered questions were treated as missing data during statistical analysis. Complete case analysis was performed as a sensitivity analysis to investigate whether or not the main results were consistent with alternatives.

### 2.5. Ethical Approval

This study was approved by the Institutional Review Board of Daejeon Korean Medicine Hospital, Daejeon University (IRB No. DJDSKH-19-BM-12-5), and exempted from both formal ethical review and informed consent (IRB No. DJDSKH-19-BM-12) as its procedures involved only negligible risks, participation was voluntary, and participants had received sufficient study information from the researchers.

## 3. Results

### 3.1. Participants

Of the 200 caregivers surveyed, we received responses from 196 (98%). After excluding all insufficient responses, we used 175 responses (87.5%) for analysis (Supplementary Figure 1).

### 3.2. General Characteristics of Respondents

The demographic characteristics of respondents are shown in Table 1. The respondents consisted of 153 (87.4%) females and 21 (12.0%) males. Of the 153 female respondents, 152 (86.9%) were mothers, 1 (0.6%) was a grandmother, 80 (52.6%) experienced menarche in grades 7-8 of secondary school, and 60 (39.5%) experienced menarche in grades 5-6 of primary school.

### 3.3. Awareness of PP

PP was recognized through hair and body odor (73.1%), uvula development (23.1%), and testicle development (19.2%) in boys; 41.3% caregivers of boys with PP recognized their sexual maturity. Among the girls' caregivers, 92.9% recognized their children's PP, mostly through breast lump development (79.8%), followed by hair and body odor (35.6%), breast itching and pain (18.3%), and sebum secretion and occurrence of acne (13.5%).

Caregivers believed that the major causes of PP were as follows: exposure to environmental hormones (60.7%), followed by westernized eating habits (53.6%), obesity (32.7%), use of smart devices (21.4%), and stress (7.7%). Heredity, premature birth, and intellectual maturity were also identified as other causes.

### 3.4. Acquisition of Information about PP

**Figure 1:**
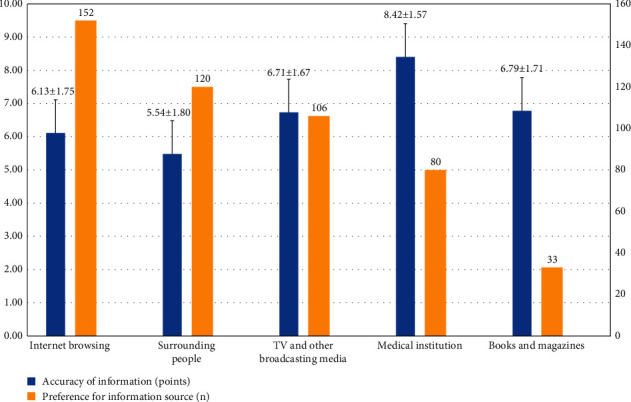
Preferences for information source and accuracy of information. Blue: accuracy of precocious puberty; yellow: preference for an information source.

In this study, 86.9% of caregivers sought most of their information about PP from the Internet. However, they believed that it had a low accuracy (6.11 ± 1.79 points on a 10-point scale). Nevertheless, respondents trusted information from medical institutions the most (8.40 ± 1.56 points) (Figure 1).

### 3.5. Perception of Treatment for PP

Of the total respondents, 98 had no treatment experience, whereas 77 had different treatment experiences: 27 had experience with CT, 39 with TKM, and 11 with both treatments.

The total preference for CT was 16.0%, with 40.7% of the caregivers with CT experience preferring it. The total preference for TKM was 21.1%, with 46.2% of the caregivers with TKM experience preferring it. Among the caregivers with no treatment experience, 16.3% preferred CT and 14.3% preferred TKM. HM was the option most favoured by 62.3% of the total participants, comprising 48.1%, 53.8%, and 63.6% from the CT, TKM, and both treatment groups, respectively, whereas 68.4% preferred HM only when they did not have treatment experience. The Chi-square test results revealed a statistically significant difference in treatment preferences according to the treatment experience (*p* ≤ 0.001), and the same result was found when analysis after listwise deletion was performed (*p* ≤ 0.001).

Among TKM methods, expectations of the effectiveness of herbal medicine were the highest (6.05 ± 1.97 points on a 10-point scale), rather than acupuncture or moxibustion.

Expectations of effectiveness were highest in HM (7.70 ± 1.61 points), followed by CT (6.75 ± 1.65 points) and TKM (6.05 ± 2.09 points). An examination of the expectations of the effectiveness according to the treatment experience showed that CT was highest in the group with CT experience (7.11 ± 1.45 points) and TKM was highest in the group with TKM experience (7.03 ± 1.77 points). While expectations of the effectiveness of TKM showed significant differences according to the treatment experience (*F* = 4.566, *p*=0.004), CT and HM did not show significant differences (*p* > 0.05). Expectations of the effectiveness of TKM were higher in the group with TKM experience than among the CT and nontreatment groups (Supplementary Table A1). In the complete case analysis, expectations of the effectiveness of TKM also showed significant differences according to the treatment experience (*F* = 3.031, *p*=0.031), whereas CT and HM did not. More details are available in Supplementary Table B1.

### 3.6. Concerns about PP

The mean stress of caregivers due to PP was 5.75 ± 2.92 points on a 10-point scale. Moreover, the score was statistically significantly higher if they had previous experience in treating PP (6.87 ± 2.23 points for the experience group, and 4.72 ± 3.24 points for the nonexperience group; *t* = 4.877, *p* < 0.05).

The CT group had the highest stress levels (7.48 ± 2.31 points) compared to the TKM group (6.62 ± 2.16 points) and groups with both treatments (6.36 ± 2.13 points). There was a statistically significant difference in the TKM and CT groups compared to the nonexperience group (*F* = 8.294, *p* ≤ 0.001; Supplementary Table A2). As a result of complete case analysis, the TKM and CT groups showed significant differences compared to the nonexperience group (*F* = 7.146, *p* ≤ 0.001; Supplementary Table B2).

Caregivers' concerns about PP were as follows: short stature (81.7%), early menarche (46.9%), and adolescent emotional problems (32.0%). In addition, early development of SSC was the most commonly reported reason for visiting medical institutions (47.4%), followed by short stature (31.6%).

The main concerns among caregivers regarding CT for PP were potential side effects of treatment (61.1%), followed by high treatment costs (45.1%), treatment effectiveness (44.6%), and the potential for overtreatment (20.0%). In addition, caregivers were concerned that the children would get hurt, treatment outcomes would not meet their expectations, and treatment would not be worth the time and cost expended (Table 2).

### 3.7. Awareness of HM and Children's Actual Habits

The three areas of diet, exercise, and smart device usage were investigated for HM.

Overeating (48.9%) was the most common eating habit that caregivers perceived as affecting PP, followed by frequent eating out (47.1%), imbalanced diet (44.3%), and irregular mealtimes (33.3%).

Foods that were perceived as affecting PP were “instant food and fast food” (73.0%), “sugar content food (chocolate, candy, etc.)” (33.9%), “greasy food” (27.6%), and “beans” (21.8%). The children's favourite foods were “meats” (56.6%), “sugar content foods” (33.9%), and “instant food and fast food” (19.1%). About 43% of respondents' children ate foods perceived as affecting PP, of which “instant food and fast food” were the most common.

The respondents reported an association between PP and exercise. Furthermore, 89% of respondents chose aerobic exercise as a preventive measure for PP. Aerobic exercise was also the most common exercise that the respondents' children engaged in (61.8%). Although about 20% of respondents recognized the need for aerobic exercise, they did not actually participate or enforce any type of exercise. The average frequency of exercise for preventing PP, as recognized by caregivers, was 4.35 ± 1.81 days, but 64.6% of the respondents' children exercised for fewer days.

**Figure 2:**
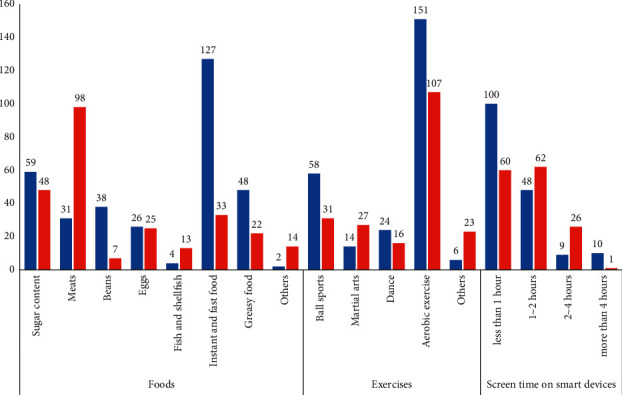
Comparison between habits perceived as affecting precocious puberty and children's actual habits (multiple answers permitted). Blue: the habits (foods, exercises, and screen time on smart devices) perceived as affecting precocious puberty; red: children's actual habits (foods, exercises, and screen time on smart devices).

Most caregivers reported a relationship between screen time on smart devices and PP; moreover, they believed that smart device usage should be restricted to prevent PP. While 57.1% of caregivers recognized that smart devices should be restricted to less than 1 hour per day, their children often used smart devices for longer durations (41.4%) (Figure 2).

## 4. Discussion

This study was conducted to identify caregivers' awareness of PP and their perceptions regarding CT and CAM and provide a basis for establishing more appropriate treatment approaches for children with PP. Respondents recognized their children's PP through breast lump development, as well as hair and body odors. As regards the treatment approach for PP, caregivers preferred HM, followed by TKM and CT. However, they experienced trouble managing their children's habits and were having difficulties obtaining accurate information. Respondents expressed concern about potential side effects and the high cost of treatment for PP.

### 4.1. Awareness of PP

Most of the respondents recognized PP among girls, while only half were able to recognize it among boys. In general, detecting PP in girls is simpler as expressions of SSC, including breast development, can be easily identified [22]. However, it is difficult to observe the signs of SSC, such as testicle enlargement, in boys. Moreover, it is often difficult to detect PP among boys because of the perception that boys mature in the later stages of adolescence. Therefore, patients with PP in Korea often fail to receive timely treatment [23]. It seems that there is a need to raise awareness amongst caregivers to enable them to identify SSC in boys more quickly.

Hair and body odors are important early signs of SSC, and despite being recognizable symptoms, they have been overlooked in many studies [24]. As the respondents were aware that PP could be identified through hair and body odors, it suggests that caregivers have awareness and a high level of knowledge about the early signs of SSC. The caregivers recognized the cause of PP consistent with results from recent epidemiological studies. According to Althobaiti et al. [25], higher education levels were associated with higher knowledge of the disease. Thus, this study may have had potential biases in responses, given most participants had high education levels. Further studies need to be conducted with respondents from various educational backgrounds.

### 4.2. Acquisition of Information about PP

Most of the respondents (86.9%) obtained information about PP from the Internet owing to the lack of reliable sources for information acquisition. The treatment of children's diseases depends entirely on the choices of caregivers, who try to gather as much information as possible on treatment alternatives [17]. However, even after deciding on the treatment, caregivers tend to search for additional information and become anxious because they are unsure about what information to trust [16]. According to Lee et al., caregivers responded that providing accurate information on PP needs to be done at the national level [17]. Therefore, it is necessary to develop a way for caregivers to obtain accurate information from the government and medical institutions.

### 4.3. Perception of Treatment for PP

Among the three treatment approaches, the highest preference was for HM (61.7%), followed by TKM (21.1%) and CT (16%). Caregivers' perceptions and reluctance to avail invasive or artificial treatment may have affected these results. In addition, the results may have been affected by easier access to information about HM than professional CT or TKM methods. Compared to HM, which has high expectations for treatment and preferences, CT has relatively high expectations related to its effectiveness, but lowest preference. Concerns about potential side effects of hormone therapy [16] and feeling sorry for children who are suffering from the treatment [17] may have affected these results. If a specific treatment is required, it is necessary to help caregivers feel safe by explaining, in detail, the effectiveness and safety of the treatment. Most results on the comparisons of expectations of the effectiveness according to the treatment experience showed no significant differences. Only the expectations for TKM showed a significant difference compared to the TKM experience group and the nontreatment group. Vague expectations of TKM and good memories from previous experiences may have affected this. Further research is needed on what factors led to these results. In treating PP, clinicians must be aware of the effects and side effects of CT and CAM (including TKM) and should be able to present treatment alternatives according to caregivers' perceptions and preferences.

### 4.4. Concerns about PP

Previous reports revealed that children's diseases and disorders increase caregivers' stress levels, while also negatively affecting their quality of life [26, 27]. Interestingly, caregivers of patients with PP often feel guilty [21] and experience heightened stress levels [22]. In this study, caregivers with previous experience in treating their children's PP also experienced high stress levels. This finding provides further confirmation that PP confers additional stress on caregivers. Furthermore, stress level was found to be the highest in the CT group, which is thought to be the effect of concerns about CT, its side effects, and costs.

The CT for PP is GnRHa, with most worrisome side effect being the ultimate attenuation of height in adulthood. Several studies have shown that adult height decreases after GnRHa treatment in children with central PP [28–30]. Moreover, weight gain, decrease in bone density, psychological atrophy, depression, minor headaches, nausea, and facial flushing are reported, further increasing caregivers' concerns [30]. Recently, several studies have been published, in which CAMs such as TKM have effects that are similar to CT, but safer. According to Shim et al., a two-year herbal medicine treatment effectively increased the duration of breast lump development to the menarche of patients with PP and showed an improvement in their growth rates [15]. Several studies conducted in China reported that traditional Chinese medicines were also effective in improving the predicted adult height of patients with idiopathic PP [31, 32], or caregivers' concerns about the potential side effects of CT, and TKM can be a good treatment alternative.

According to Choi and Park [23], the total cost of treating patients with GnRHa was approximately KRW 21.6 billion in 2015, a 1.9-fold increase from 2010. In Korea, health insurance is only applied if the drug is administered before the ages of 9 and 10 for girls and boys, respectively. Therefore, the cost of treatment would increase significantly in cases of delayed diagnosis. Previous research has revealed caregivers' concerns about financial burdens, with caregivers responding that expansion of insurance for PP is required at the national level [17]. The burden of care for families of patients with chronic diseases is often attributable to the time and financial constraints needed to initiate care, rather than engagement in the care process itself [33]. With 45.1% of caregivers concerned about the high treatment costs, clinicians should be well aware of these concerns and be able to suggest alternatives in consideration of these concerns.

### 4.5. Awareness of HM and Children's Actual Habits

Previous studies have revealed that the frequency of eating out, amount of meat intake, and obesity have a significant impact on PP. Moreover, increased screen time on smart devices can also be a trigger for PP [34]. While the study respondents were aware of this, they did not effectively restrict their children's eating habits or screen time on smart devices. In addition, exercise is effective in weight control and body fat reduction, as well as in controlling the secretion of female hormones [35]. Therefore, appropriate exercise should be implemented. However, caregivers in this study did not educate their children on how to exercise, indicating that they require support in managing their children's habits. While respondents trusted information from medical institutions the most, medical counselling often had a greater influence on HM than caregivers.

Therefore, when planning treatment for PP, clinicians' direct education on regular exercise and balanced nutrition should be included in the treatment plans.

### 4.6. Limitations

This study also had some limitations. We conducted the survey only at two TKM institutions, and the number of respondents was limited to 175. Therefore, it is unreasonable to generalize the research results to the entire population. In the process of developing the survey questionnaire, while expert reviews were conducted to increase the validity of the tool, additional pretesting, including cognitive interviewing and pilot testing, was not conducted. Therefore, we were unable to confirm how the survey would function in relation to real people, which may have resulted in a response error. In addition, although we conducted reliability analysis to evaluate internal consistency, we could not fully assess the reliability because we could not appraise the consistency between raters and test-retest reliability. In future studies, a preliminary evaluation that increases the reliability and validity of the tool should be sufficiently conducted. In this study, reporting heterogeneity may have occurred because the responses were different according to the respondents' characteristics (demographic characteristics and medical institution experiences). Furthermore, information bias, such as recall bias or memory loss bias, could have occurred because this study was a self-reporting survey that included treatment experience and respondents' awareness. Furthermore, the possibility of interviewer bias cannot be ruled out as the survey was conducted by a TKM institution. Consequently, the results of the survey may have been overestimated or underestimated. In future research, methods for correcting the reporting heterogeneity, such as the anchoring vignette method, should be applied.

However, this study is significant in that it is the first study that used a survey to investigate caregivers' perceptions of PP and their preferences for its treatment. In addition, its results can be used as basic data for planning treatment for PP.

## 5. Conclusions

The caregivers of children with PP had relatively detailed knowledge about the causes and symptoms of PP, but they were having some difficulty obtaining reliable information. In addition, they preferred prevention and management of the condition through HM rather than artificial treatment, such as hormonal treatment or CAM, and were also having difficulty managing their children's habits. This study identified key considerations in developing treatment plans for patients with PP. Its results could be used to establish CAM treatment plans for children with PP and consult their caregivers. Considering these results, clinicians should try to establish a more appropriate treatment plan for children with PP.

Further studies should consider the following: a multicentre survey study, including both CT and TKM institutions, investigations into CAM providers' perceptions regarding PP, and the development and efficacy evaluation of treatment programmes based on this study's results.

## Figures and Tables

**Table 1:** Demographic characteristics of respondents.

Classification	Total (*n* = 175)	
	N	%
Sex		
Male	21	12.0
Female	153	87.4

Relationship		
Father	21	12.0
Mother	152	86.9
Others	1	0.6

Age		
25–29 years	2	1.1
30–34 years	7	4.0
35–39 years	42	24.0
40–45 years	89	50.9
Older than 45 years	34	19.4

Education levels		
High school	21	12.0
Bachelor's degree	111	63.4
Master's degree	42	24.0

Employment status		
Employed	117	66.9
Unemployed	57	32.6

Menarche of mothers		
Elementary school grades 3–4	2	1.3
Elementary school grades 5–6	60	39.5
Middle school grades 1–2	80	52.6
Above middle school grade 3	10	6.6

**Table 2:** Parental perceptions of precocious puberty.

Questions	*N*	%
1. What symptoms of your child made you suspect precocious puberty? (for girls)		
Development of a breast lump	83	79.8
Itching breast or occurrence of breast pain, in the event of a slight bump	19	18.3
Sebum secretion and occurrence of acne	14	13.5
Occurrence of hair and body odor	37	35.6
Pubic or underarm hair	12	11.5
Vaginal discharge	3	2.9

2. What symptoms of your child made you suspect precocious puberty? (for boys)		
Testicle development	5	19.2
The penis gets longer, and the color of the penis changes	3	11.5
Sebum secretion and occurrence of acne	3	11.5
Occurrence of hair and body odor	19	73.1
Pubic or underarm hair	2	7.7
Uvula develops, and the voice starts to change	6	23.1

3. Major causes of precocious puberty		
Westernized eating habits	90	53.6
Exposure to environmental hormones	102	60.7
Use of smart devices (Electromagnetic waves due to overuse of smartphones and TVs)	36	21.4
Obesity	55	32.7
Stress	13	7.7

4. Preference for information source		
Television and other broadcasting media	106	60.6
Internet browsing	152	86.9
Books and magazines	33	18.9
Surrounding people	120	68.6
Medical institutions	80	45.7

5. Previous treatment experience		
Yes	77	44
Traditional Korean medicine treatment	39	22.3
Conventional treatment	27	15.4
Both treatments	11	6.3
No	98	56

6. Preference for treatment		
Traditional Korean medicine treatment	37	21.1
Conventional treatment	28	16
Habits management	108	61.7
No treatment required	2	1.1

7. Reasons for visiting medical institutions		
Height (Short stature)	24	31.6
Early secondary sexual characteristics	36	47.4
Obesity	4	5.3
Abnormal findings at school health screening and infant health screening	1	1.3

8. Concerns about precocious puberty		
Height (Short stature)	143	81.7
Early menarche	82	46.9
Academic performance	7	4.0
Adolescent emotional problems	56	32.0

9. Concerns about treatment		
Potential side effects of treatment	107	61.1
High treatment costs	79	45.1
Doubts about treatment effectiveness	78	44.6
Potential for overtreatment	35	20.0

10. Eating habits perceived as affecting precocious puberty		
Imbalanced diet	77	44.3
Overeating	85	48.9
Light eating	2	1.1
Irregular mealtimes	58	33.3
Frequent eating out	82	47.1

11. Comparison between food perceived as affecting precocious puberty and children's actual habits		
Eating foods perceived as affecting precocious puberty	76	43.4
Sugar content food (chocolate, candy, etc.)	20	26.3
Meats	15	19.7
Beans	2	2.6
Eggs (eggs, quail eggs, pollack roe, etc.)	5	6.6
Instant and fast foods	25	32.9
Greasy foods (deep-fried food, pancakes, etc.)	8	10.5
Not eating foods perceived as affecting precocious puberty	99	56.6

12. Comparison between exercise perceived as preventing precocious puberty and children's actual habits		
Perform the same types of exercise	131	74.9
Ball sports (such as basketball and football)	16	12.2
Martial arts (such as Taekwondo and Hapkido)	5	3.8
Dance (such as ballet and broadcasting dance)	7	5.3
Aerobic exercise (such as walking, running, and skipping rope)	90	68.7
Perform different types of exercises	38	21.7

13. Comparison between frequency of exercise perceived as preventing precocious puberty and children's actual habits		
Exercise less than perceived frequency	113	64.6
Exercise equal to perceived frequency	38	21.7
Exercise more than perceived frequency	20	12.4

14. Comparison between screen time on smart devices perceived as affecting precocious puberty and children's actual habits		
Usage less than perceived screen time	22	12.6
Usage equal to perceived screen time	79	45.1
Usage more than perceived screen time	72	41.1

## Data Availability

Data will be made available upon reasonable request to the corresponding author.

## Supplementary Materials

Supplementary Materials (1) Survey form; (2) Supplementary Figure 1: enrolment flow chart; (3) Supplementary tables: (a) Supplementary Table A1: expectations of the effectiveness of treatment according to the treatment experiences, (b) Supplementary Table A2: stress level of caregivers according to the treatment experiences, (c) Supplementary Table B1: expectations of the effectiveness of treatment according to the treatment experiences through complete case analysis, and (d) Supplementary Table B2: stress level of caregivers according to the treatment experiences through complete case analysis.
