# Supplementary material for: Perceptions and Treatment of Precocious Puberty: A Questionnaire Survey among Caregivers in South Korea
Source: Evid Based Complement Alternat Med. 2022 Jul 21;2022:9413188. doi: 10.1155/2022/9413188 (PMC9334087; doi:10.1155/2022/9413188)
Supplement: Supplementary Materials — (1) Survey form; (2) Supplementary Figure 1: enrolment flow chart; (3) Supplementary tables: (a) Supplementary Table A1: expectations of the effectiveness of treatment according to the treatment experiences, (b) Supplementary Table A2: stress level of caregivers according to the treatment experiences, (c) Supplementary Table B1: expectations of the effectiveness of treatment according to the treatment experiences through complete case analysis, and (d) Supplementary Table B2: stress level of caregivers according to the treatment experiences through complete case analysis. [file 9413188.f1.zip › supplement; survey form210723.docx]

A survey on parent’s illness perceptions of precocious puberty

Ⅰ. General characteristics

1. What is your gender?

(1) Male (2) Female

2. What is the relationship between you and your child?

(1) Mother (2) Father (3) Grandmother

(4) Grandfather (5) Other ( )

3. How old are you?

(1) 20–24 years old (2) 25–29 years old (3) 30–34 years old

(4) 35–39 years old (5) 40–45 years old (6) 45 years old or older

4. What is your education level?

(1) Primary school or lower

(2) Secondary school

(3) University graduate

(4) Graduate school graduate

5. Are you currently employed?

(1) Yes

(2) No

6. When was your menarche?

(1) Year 2 or lower of primary school

(2) Year 3-4 of primary school

(3) Year 5-6 of primary school

(4) Year 7-8 of secondary school

(5) Year 9 or higher of secondary school

7. How many children do you currently have?

(1) One (2) Two (3) Three (4) Four or more

8. How old is your child? (please complete for all children.)

1) _____ years of age 2) _____ years of age 3) _____ years of age 4) _____ years of age

**Ⅱ. Survey on the perception of information acquisition for precocious puberty**

1. Precocious puberty refers to a secondary sexual development in girls (chest development) before the age of eight and boys (testicular development) before the age of nine. How much do you know about the definition of precocious puberty?

| I do not know well | |  | |  | |  | |  | |  | |  | |  | |  | |  | |  | | I know very well | |
| --- | --- | --- | --- | --- | --- | --- | --- | --- | --- | --- | --- | --- | --- | --- | --- | --- | --- | --- | --- | --- | --- | --- | --- |
|  |  |  | |  | |  | |  | |  | |  | |  | |  | |  | |  | |  |  |
|  |  |  | |  | |  | |  | |  | |  | |  | |  | |  | |  | |  |  |
|  |  |  | |  | |  | |  | |  | |  | |  | |  | |  | |  | |  |  |
|  | 0 | | 1 | | 2 | | 3 | | 4 | | 5 | | 6 | | 7 | | 8 | | 9 | | 10 | |  |

2. Where do you usually get information about precocious puberty? (please enter the number.)

1) 1st ( ) 2) 2nd ( ) 3) 3rd ( )

(1) Television and other broadcasting media

(2) Internet browsing

(3) Books and magazines

(4) People around you

(5) Medical institutions

(6) Other ( )

3. To what extent do you think infomation about precocious puberty obtained from each media is accurate?

3-1. Television and other broadcasting media

| Not accurate | |  | |  | |  | |  | |  | |  | |  | |  | |  | |  | | Very accurate | |
| --- | --- | --- | --- | --- | --- | --- | --- | --- | --- | --- | --- | --- | --- | --- | --- | --- | --- | --- | --- | --- | --- | --- | --- |
|  |  |  | |  | |  | |  | |  | |  | |  | |  | |  | |  | |  |  |
|  |  |  | |  | |  | |  | |  | |  | |  | |  | |  | |  | |  |  |
|  |  |  | |  | |  | |  | |  | |  | |  | |  | |  | |  | |  |  |
|  | 0 | | 1 | | 2 | | 3 | | 4 | | 5 | | 6 | | 7 | | 8 | | 9 | | 10 | |  |

3-2. Internet browsing

| Not accurate | |  | |  | |  | |  | |  | |  | |  | |  | |  | |  | | Very accurate | |
| --- | --- | --- | --- | --- | --- | --- | --- | --- | --- | --- | --- | --- | --- | --- | --- | --- | --- | --- | --- | --- | --- | --- | --- |
|  |  |  | |  | |  | |  | |  | |  | |  | |  | |  | |  | |  |  |
|  |  |  | |  | |  | |  | |  | |  | |  | |  | |  | |  | |  |  |
|  |  |  | |  | |  | |  | |  | |  | |  | |  | |  | |  | |  |  |
|  | 0 | | 1 | | 2 | | 3 | | 4 | | 5 | | 6 | | 7 | | 8 | | 9 | | 10 | |  |

3-3. Books and magazines

| Not accurate | |  | |  | |  | |  | |  | |  | |  | |  | |  | |  | | Very accurate | |
| --- | --- | --- | --- | --- | --- | --- | --- | --- | --- | --- | --- | --- | --- | --- | --- | --- | --- | --- | --- | --- | --- | --- | --- |
|  |  |  | |  | |  | |  | |  | |  | |  | |  | |  | |  | |  |  |
|  |  |  | |  | |  | |  | |  | |  | |  | |  | |  | |  | |  |  |
|  |  |  | |  | |  | |  | |  | |  | |  | |  | |  | |  | |  |  |
|  | 0 | | 1 | | 2 | | 3 | | 4 | | 5 | | 6 | | 7 | | 8 | | 9 | | 10 | |  |

3-4. People around you

| Not accurate | |  | |  | |  | |  | |  | |  | |  | |  | |  | |  | | Very accurate | |
| --- | --- | --- | --- | --- | --- | --- | --- | --- | --- | --- | --- | --- | --- | --- | --- | --- | --- | --- | --- | --- | --- | --- | --- |
|  |  |  | |  | |  | |  | |  | |  | |  | |  | |  | |  | |  |  |
|  |  |  | |  | |  | |  | |  | |  | |  | |  | |  | |  | |  |  |
|  |  |  | |  | |  | |  | |  | |  | |  | |  | |  | |  | |  |  |
|  | 0 | | 1 | | 2 | | 3 | | 4 | | 5 | | 6 | | 7 | | 8 | | 9 | | 10 | |  |

3-5. Medical institutions

| Not accurate | |  | |  | |  | |  | |  | |  | |  | |  | |  | |  | | Very accurate | |
| --- | --- | --- | --- | --- | --- | --- | --- | --- | --- | --- | --- | --- | --- | --- | --- | --- | --- | --- | --- | --- | --- | --- | --- |
|  |  |  | |  | |  | |  | |  | |  | |  | |  | |  | |  | |  |  |
|  |  |  | |  | |  | |  | |  | |  | |  | |  | |  | |  | |  |  |
|  |  |  | |  | |  | |  | |  | |  | |  | |  | |  | |  | |  |  |
|  | 0 | | 1 | | 2 | | 3 | | 4 | | 5 | | 6 | | 7 | | 8 | | 9 | | 10 | |  |

4. How easy is it to understand information about precocious puberty obtained from each media?

| Difficult to understand | |  | |  | |  | |  | |  | |  | |  | |  | |  | |  | | Easy to understand | |
| --- | --- | --- | --- | --- | --- | --- | --- | --- | --- | --- | --- | --- | --- | --- | --- | --- | --- | --- | --- | --- | --- | --- | --- |
|  |  |  | |  | |  | |  | |  | |  | |  | |  | |  | |  | |  |  |
|  |  |  | |  | |  | |  | |  | |  | |  | |  | |  | |  | |  |  |
|  |  |  | |  | |  | |  | |  | |  | |  | |  | |  | |  | |  |  |
|  | 0 | | 1 | | 2 | | 3 | | 4 | | 5 | | 6 | | 7 | | 8 | | 9 | | 10 | |  |

**Ⅲ. General characteristics of children with precocious puberty**

1. This is a survey on children with precocious puberty. Please respond for **all** of your children.

- The first time you recognised precocious puberty of your child: ___(month) (year)

- Date of birth of your child: ___(month) (year)

- Gender of your child: (1) Male (2) Female

- Current height & weight of your child: cm, kg

- The first time you recognised precocious puberty of your child: ___(month) (year)

- Date of birth of your child: ___(month) (year)

- Gender of your child: (1) Male (2) Female

- Current height & weight of your child: cm, kg

2. What symptoms of your child did you suspect precocious puberty? (for girls) (please choose up to two responses)

(1) Breast lump has developed

(2) Breast is itching, or occurrence of breast pain if bumped slightly

(3) Sebum secretion and occurrence of acne

(4) The odour of hair and sweat begins to occur

(5) Pubic or underarm hair

(6) Vaginal discharge

3. What symptoms of your child did you suspect precocious puberty? (for boys) (please choose up to two responses)

(1) The testicles develop to get bigger

(2) The penis gets longer, and the colour of the penis changes

(3) Sebum is secreted, and acne occurs

(4) The odour of hair and sweat begins to occur

(5) Pubic or underarm hair

(6) Uvula develops, and the voice starts to change

4. What do you think is the cause of your child's precocious puberty? (please choose up to two responses)

(1) Westernised eating habits

(2) Exposure to environmental hormones

(3) Use of smart devices (Electromagnetic waves due to overuse of smartphones and TVs)

(4) Obesity

(5) Stress

(6) Other ( )

Ⅳ. Awareness survey on the treatment of precocious puberty

1. What treatment do you think is necessary for precocious puberty?

(1) Korean medicine treatment

(2) Western medicine treatment

(3) Exercise and life management

(4) No treatment required

(5) Other ( )

2. If your child has ever been treated for precocious puberty, where did she/he receive the treatment? (multiple responses available)

(1) Korean medicine hospital

(2) Korean medicine clinic

(3) General hospital

(4) Clinics (pediatric clinic, family medicine clinic, etc.)

(5) Other ( )

2-1. If your child is being treated for precocious puberty, how long has it been since the treatment started?

It has been ( ) year (s) ( ) month(s) since it started

3. What made your child visit the medical institution for the first time due to precocious puberty?

(1) Height (short stature)

(2) Early secondary sexual characteristics (breast development, menarche start, testicular development):

(3) Obesity

(4) Abnormal findings at school health screening and infant health screening:

(5) Other ( )

4. What is your reliability in effectiveness for each of the following treatments?

4-1. Korean medicine treatment

| I do not trust | |  | |  | |  | |  | |  | |  | |  | |  | |  | |  | | I trust a lot | |
| --- | --- | --- | --- | --- | --- | --- | --- | --- | --- | --- | --- | --- | --- | --- | --- | --- | --- | --- | --- | --- | --- | --- | --- |
|  |  |  | |  | |  | |  | |  | |  | |  | |  | |  | |  | |  |  |
|  |  |  | |  | |  | |  | |  | |  | |  | |  | |  | |  | |  |  |
|  |  |  | |  | |  | |  | |  | |  | |  | |  | |  | |  | |  |  |
|  | 0 | | 1 | | 2 | | 3 | | 4 | | 5 | | 6 | | 7 | | 8 | | 9 | | 10 | |  |

4-2. Western medicine treatment

| I do not trust | |  | |  | |  | |  | |  | |  | |  | |  | |  | |  | | I trust a lot | |
| --- | --- | --- | --- | --- | --- | --- | --- | --- | --- | --- | --- | --- | --- | --- | --- | --- | --- | --- | --- | --- | --- | --- | --- |
|  |  |  | |  | |  | |  | |  | |  | |  | |  | |  | |  | |  |  |
|  |  |  | |  | |  | |  | |  | |  | |  | |  | |  | |  | |  |  |
|  |  |  | |  | |  | |  | |  | |  | |  | |  | |  | |  | |  |  |
|  | 0 | | 1 | | 2 | | 3 | | 4 | | 5 | | 6 | | 7 | | 8 | | 9 | | 10 | |  |

4-3. Habit management

| I do not trust | |  | |  | |  | |  | |  | |  | |  | |  | |  | |  | | I trust a lot | |
| --- | --- | --- | --- | --- | --- | --- | --- | --- | --- | --- | --- | --- | --- | --- | --- | --- | --- | --- | --- | --- | --- | --- | --- |
|  |  |  | |  | |  | |  | |  | |  | |  | |  | |  | |  | |  |  |
|  |  |  | |  | |  | |  | |  | |  | |  | |  | |  | |  | |  |  |
|  |  |  | |  | |  | |  | |  | |  | |  | |  | |  | |  | |  |  |
|  | 0 | | 1 | | 2 | | 3 | | 4 | | 5 | | 6 | | 7 | | 8 | | 9 | | 10 | |  |

5. Expected effectiveness of Korean medicine treatment methods

5-1. Acupuncture

| It does not work at all | |  | |  | |  | |  | |  | |  | |  | |  | |  | |  | | It works very well | |
| --- | --- | --- | --- | --- | --- | --- | --- | --- | --- | --- | --- | --- | --- | --- | --- | --- | --- | --- | --- | --- | --- | --- | --- |
|  |  |  | |  | |  | |  | |  | |  | |  | |  | |  | |  | |  |  |
|  |  |  | |  | |  | |  | |  | |  | |  | |  | |  | |  | |  |  |
|  |  |  | |  | |  | |  | |  | |  | |  | |  | |  | |  | |  |  |
|  | 0 | | 1 | | 2 | | 3 | | 4 | | 5 | | 6 | | 7 | | 8 | | 9 | | 10 | |  |

5-2. Herbal medicine

| It does not work at all | |  | |  | |  | |  | |  | |  | |  | |  | |  | |  | | It works very well | |
| --- | --- | --- | --- | --- | --- | --- | --- | --- | --- | --- | --- | --- | --- | --- | --- | --- | --- | --- | --- | --- | --- | --- | --- |
|  |  |  | |  | |  | |  | |  | |  | |  | |  | |  | |  | |  |  |
|  |  |  | |  | |  | |  | |  | |  | |  | |  | |  | |  | |  |  |
|  |  |  | |  | |  | |  | |  | |  | |  | |  | |  | |  | |  |  |
|  | 0 | | 1 | | 2 | | 3 | | 4 | | 5 | | 6 | | 7 | | 8 | | 9 | | 10 | |  |

5-3. Moxibustion treatment

| It does not work at all | |  | |  | |  | |  | |  | |  | |  | |  | |  | |  | | It works very well | |
| --- | --- | --- | --- | --- | --- | --- | --- | --- | --- | --- | --- | --- | --- | --- | --- | --- | --- | --- | --- | --- | --- | --- | --- |
|  |  |  | |  | |  | |  | |  | |  | |  | |  | |  | |  | |  |  |
|  |  |  | |  | |  | |  | |  | |  | |  | |  | |  | |  | |  |  |
|  |  |  | |  | |  | |  | |  | |  | |  | |  | |  | |  | |  |  |
|  | 0 | | 1 | | 2 | | 3 | | 4 | | 5 | | 6 | | 7 | | 8 | | 9 | | 10 | |  |

Ⅴ. Survey on stress due to precocious puberty

1. How stressed do you think your child is due to precocious puberty?

| Not at all | |  | |  | |  | |  | |  | |  | |  | |  | |  | |  | | Very severe | |
| --- | --- | --- | --- | --- | --- | --- | --- | --- | --- | --- | --- | --- | --- | --- | --- | --- | --- | --- | --- | --- | --- | --- | --- |
|  |  |  | |  | |  | |  | |  | |  | |  | |  | |  | |  | |  |  |
|  |  |  | |  | |  | |  | |  | |  | |  | |  | |  | |  | |  |  |
|  |  |  | |  | |  | |  | |  | |  | |  | |  | |  | |  | |  |  |
|  | 0 | | 1 | | 2 | | 3 | | 4 | | 5 | | 6 | | 7 | | 8 | | 9 | | 10 | |  |

2. How stressed are you by your child's precocious puberty?

| Not at all | |  | |  | |  | |  | |  | |  | |  | |  | |  | |  | | Very severe | |
| --- | --- | --- | --- | --- | --- | --- | --- | --- | --- | --- | --- | --- | --- | --- | --- | --- | --- | --- | --- | --- | --- | --- | --- |
|  |  |  | |  | |  | |  | |  | |  | |  | |  | |  | |  | |  |  |
|  |  |  | |  | |  | |  | |  | |  | |  | |  | |  | |  | |  |  |
|  |  |  | |  | |  | |  | |  | |  | |  | |  | |  | |  | |  |  |
|  | 0 | | 1 | | 2 | | 3 | | 4 | | 5 | | 6 | | 7 | | 8 | | 9 | | 10 | |  |

3. What are your concerns concerns about your child's precocious puberty?? (please choose up to two responses)

(1) Height (short stature)

(2) Early menarche

(3) Academic performance

(4) Adolescent emotional problem

(5) Other ( )

4. What concerns do you have when your child is being treated for precocious puberty? (Please choose up to two responses)

(1) Potential side effects of the treatment

(2) High treatment cost

(3) Doubt about the effectiveness of treatment

(4) Potential for overtreatment

(5) Other ( )

Ⅵ. Survey on the level of perception of precocious puberty - Awareness of life management

1. How much do you know about the life management of precocious puberty?

| I do not know at all | |  | |  | |  | |  | |  | |  | |  | |  | |  | |  | | I know very well | |
| --- | --- | --- | --- | --- | --- | --- | --- | --- | --- | --- | --- | --- | --- | --- | --- | --- | --- | --- | --- | --- | --- | --- | --- |
|  |  |  | |  | |  | |  | |  | |  | |  | |  | |  | |  | |  |  |
|  |  |  | |  | |  | |  | |  | |  | |  | |  | |  | |  | |  |  |
|  |  |  | |  | |  | |  | |  | |  | |  | |  | |  | |  | |  |  |
|  | 0 | | 1 | | 2 | | 3 | | 4 | | 5 | | 6 | | 7 | | 8 | | 9 | | 10 | |  |

1 Eating habits

1. How much do you think is related to eating habits and precocious puberty?

| It is not relevant at all | |  | |  | |  | |  | |  | |  | |  | |  | |  | |  | | It is very relevant | |
| --- | --- | --- | --- | --- | --- | --- | --- | --- | --- | --- | --- | --- | --- | --- | --- | --- | --- | --- | --- | --- | --- | --- | --- |
|  |  |  | |  | |  | |  | |  | |  | |  | |  | |  | |  | |  |  |
|  |  |  | |  | |  | |  | |  | |  | |  | |  | |  | |  | |  |  |
|  |  |  | |  | |  | |  | |  | |  | |  | |  | |  | |  | |  |  |
|  | 0 | | 1 | | 2 | | 3 | | 4 | | 5 | | 6 | | 7 | | 8 | | 9 | | 10 | |  |

2. What eating habits do you think affect precocious puberty? (please choose up to two responses)

(1) Imbalanced diet

(2) Overeating

(3) Light eating

(4) Irregular mealtime

(5) Frequent eating out

(6) Other ( )

3. What foods do you think affect precocious puberty? (please choose up to two responses)

(1) Sugar content food (chocolate, candy, etc.)

(2) Meats

(3) Beans

(4) Eggs (eggs, quail eggs, pollack roe, etc.)

(5) Fish and shellfish

(6) Instant food and fast food

(7) Greasy food (deep-fried food, pancake, etc.)

(8) Other ( )

2 Exercise

1. How much do you think exercise is related to precocious puberty?

| It is not relevant at all | |  | |  | |  | |  | |  | |  | |  | |  | |  | |  | | It is very relevant | |
| --- | --- | --- | --- | --- | --- | --- | --- | --- | --- | --- | --- | --- | --- | --- | --- | --- | --- | --- | --- | --- | --- | --- | --- |
|  |  |  | |  | |  | |  | |  | |  | |  | |  | |  | |  | |  |  |
|  |  |  | |  | |  | |  | |  | |  | |  | |  | |  | |  | |  |  |
|  |  |  | |  | |  | |  | |  | |  | |  | |  | |  | |  | |  |  |
|  | 0 | | 1 | | 2 | | 3 | | 4 | | 5 | | 6 | | 7 | | 8 | | 9 | | 10 | |  |

2. How many days a week do you think your child should exercise to prevent and treat precocious puberty?

(1) It has nothing to do with Exercise.

(2) One day

(3) Two days

(4) Three days

(5) Four days

(6) Five days

(7) Six days

(8) Every day

3. How long per exercise do you think is necessary to prevent and treat precocious puberty?

(1) Within 10 minutes

(2) 10 to 30 minutes

(3) 30 minutes to 1-hour

(4) 1 hour or more

4. What kind of exercise do you think is good for preventing and treating precocious puberty? (please choose up to two responses)

(1) Ball sports such as basketball and football

(2) Martial arts such as Taekwondo and Hapkido

(3) Dances such as ballet and broadcasting dance

(4) Aerobic exercises such as walking, running and skipping rope

(5) Other ( )

3 Use of smart devices

1. How much do you think precocious puberty is related to the use of smart devices (television, smartphone, computer, etc.)?

| It is not relevant at all | |  | |  | |  | |  | |  | |  | |  | |  | |  | |  | | It is very relevant | |
| --- | --- | --- | --- | --- | --- | --- | --- | --- | --- | --- | --- | --- | --- | --- | --- | --- | --- | --- | --- | --- | --- | --- | --- |
|  |  |  | |  | |  | |  | |  | |  | |  | |  | |  | |  | |  |  |
|  |  |  | |  | |  | |  | |  | |  | |  | |  | |  | |  | |  |  |
|  |  |  | |  | |  | |  | |  | |  | |  | |  | |  | |  | |  |  |
|  | 0 | | 1 | | 2 | | 3 | | 4 | | 5 | | 6 | | 7 | | 8 | | 9 | | 10 | |  |

2. How much do you think smart devices should be restricted to prevent and treat precocious puberty?

(1) There is no need to restrict

(2) Up to 4 hours a day

(3) Up to 2 hours a day

(4) Up to 1 hour a day

(5) Other ( )

Ⅶ. Survey on the level of perception of precocious puberty: Life management status

1 Eating habits

1. What do you think your child's usual eating habits are?

| Very bad | |  | |  | |  | |  | |  | |  | |  | |  | |  | |  | | Very good | |
| --- | --- | --- | --- | --- | --- | --- | --- | --- | --- | --- | --- | --- | --- | --- | --- | --- | --- | --- | --- | --- | --- | --- | --- |
|  |  |  | |  | |  | |  | |  | |  | |  | |  | |  | |  | |  |  |
|  |  |  | |  | |  | |  | |  | |  | |  | |  | |  | |  | |  |  |
|  |  |  | |  | |  | |  | |  | |  | |  | |  | |  | |  | |  |  |
|  | 0 | | 1 | | 2 | | 3 | | 4 | | 5 | | 6 | | 7 | | 8 | | 9 | | 10 | |  |

1-1. If eating habits are not good, what is the problem?

( )

2. What is your child's favourite food?

(1) High-sugar content food (chocolate, candy, etc.)

(2) Meat

(3) Beans

(4) Eggs (eggs, quail eggs, pollock roe, etc.)

(5) Seafood (fish, shellfish, etc.)

(6) Instant food and fast food

(7) Greasy food (deep-fried food, pancake, etc.)

(8) Other ( )

2 Exercise

1. How many days a week does your child usually exercise?

(1) None.

(2) One day

(3) Two days

(4) Three days

(5) Four days

(6) Five days

(7) Six days

(8) Every day

2. What kind of sports does your child usually do?

(1) Ball sports such as basketball and football

(2) Martial arts such as Taekwondo and Hapkido

(3) Dance such as ballet, broadcast dance

(4) Aerobic exercises such as walking, running and skipping rope

(5) Other ( )

3 Use of smart devices

1. How long does your child usually use the smart devices per day?

(1) They do not use at all

(2) Up to 1-hour

(3) Up to 2 hours

(4) Up to 4 hours

(5) 4 hours or more

(6) Other ( )
