# Supplementary material for: Perceptions and Treatment of Precocious Puberty: A Questionnaire Survey among Caregivers in South Korea
Source: Evid Based Complement Alternat Med. 2022 Jul 21;2022:9413188. doi: 10.1155/2022/9413188 (PMC9334087; doi:10.1155/2022/9413188)
Supplement: Supplementary Materials — (1) Survey form; (2) Supplementary Figure 1: enrolment flow chart; (3) Supplementary tables: (a) Supplementary Table A1: expectations of the effectiveness of treatment according to the treatment experiences, (b) Supplementary Table A2: stress level of caregivers according to the treatment experiences, (c) Supplementary Table B1: expectations of the effectiveness of treatment according to the treatment experiences through complete case analysis, and (d) Supplementary Table B2: stress level of caregivers according to the treatment experiences through complete case analysis. [file 9413188.f1.zip › Supplementary figure 220613.docx]

**Supplementary figure 1. Enrolment flow chart**

Assessed for eligibility

(n=196)

Included

(n=175)

Excluded (n=21)

- Not responses children’s birth date (n=20)
- Not answer the questions appropriately (n=1)

Analyzed

(n=175)

Identification

Inclusion

Analysis
