# Supplementary material for: Perceptions and Treatment of Precocious Puberty: A Questionnaire Survey among Caregivers in South Korea
Source: Evid Based Complement Alternat Med. 2022 Jul 21;2022:9413188. doi: 10.1155/2022/9413188 (PMC9334087; doi:10.1155/2022/9413188)
Supplement: Supplementary Materials — (1) Survey form; (2) Supplementary Figure 1: enrolment flow chart; (3) Supplementary tables: (a) Supplementary Table A1: expectations of the effectiveness of treatment according to the treatment experiences, (b) Supplementary Table A2: stress level of caregivers according to the treatment experiences, (c) Supplementary Table B1: expectations of the effectiveness of treatment according to the treatment experiences through complete case analysis, and (d) Supplementary Table B2: stress level of caregivers according to the treatment experiences through complete case analysis. [file 9413188.f1.zip › Supplementary tables 220630.docx]

**Supplementary table A1. Expectations of the effectiveness of treatment according to the treatment experiences**

|  | N | Mean | SD | F/p-value | Post-hoc |
| --- | --- | --- | --- | --- | --- |
| Traditional Korean Medicine | | | | | |
| TKM | 39 | 7.03 | 1.77 | 4.566/0.004 | TKM>CT, None  (Scheffe) |
| CT | 26 | 5.27 | 2.44 |  |  |
| Both | 11 | 6.09 | 2.21 |  |  |
| None | 98 | 5.87 | 1.98 |  |  |
| Total(missing) | 174(1) | 6.05 | 2.09 |  |  |
| Conventional treatment | | | | | |
| TKM | 35 | 6.63 | 1.68 | 0.578/0.631 |  |
| CT | 27 | 7.11 | 1.45 |  |  |
| Both | 11 | 6.55 | 1.81 |  |  |
| None | 98 | 6.69 | 1.69 |  |  |
| Total(missing) | 171(4) | 6.74 | 1.65 |  |  |
| Habit management | | | | | |
| TKM | 39 | 7.77 | 1.61 | 0.202/0.895 |  |
| CT | 27 | 7.52 | 1.85 |  |  |
| Both | 10 | 7.50 | 1.51 |  |  |
| None | 98 | 7.73 | 1.56 |  |  |
| Total(missing) | 174(1) | 7.70 | 1.61 |  |  |

CT: conventional treatment, TKM: traditional Korean medicine

**Supplementary table A2. Stress level of caregivers according to the treatment experiences**

|  | N | Mean | SD | F/p-value | Post-hoc |
| --- | --- | --- | --- | --- | --- |
| Stress level | | | | | |
| TKM | 39 | 6.62 | 2.16 | 8.294/≤0.001 | TKM>none  CT>none  (Dunnett T3) |
| CT | 25 | 7.48 | 2.31 |  |  |
| Both | 11 | 6.36 | 2.16 |  |  |
| None | 82 | 4.72 | 2.24 |  |  |
| Total(missing) | 157(18) | 5.75 | 2.99 |  |  |

CT: conventional treatment, TKM: traditional Korean medicine

**Supplementary table B1. Expectations of the effectiveness of treatment according to the treatment experiences through complete case analysis**

|  | N | Mean | SD | F/p-value | Post-hoc |
| --- | --- | --- | --- | --- | --- |
| Traditional Korean Medicine | | | | | |
| TKM | 35 | 6.89 | 1.81 | 3.031/0.031 | TKM>CT  (Tukey HSD) |
| CT | 24 | 5.42 | 2.45 |  |  |
| Both | 10 | 6.60 | 1.51 |  |  |
| None | 82 | 5.98 | 1.98 |  |  |
| Total | 151 | 6.14 | 2.04 |  |  |
| Conventional treatment | | | | | |
| TKM | 35 | 6.63 | 1.68 | 0.468/0.705 |  |
| CT | 24 | 7.13 | 1.33 |  |  |
| Both | 10 | 6.70 | 1.83 |  |  |
| None | 82 | 6.72 | 1.75 |  |  |
| Total | 151 | 6.76 | 1.67 |  |  |
| Habit management | | | | | |
| TKM | 35 | 7.57 | 1.56 | 0.305/0.821 |  |
| CT | 24 | 7.38 | 1.91 |  |  |
| Both | 10 | 7.50 | 1.51 |  |  |
| None | 82 | 7.72 | 1.61 |  |  |
| Total | 151 | 7.62 | 1.63 |  |  |

CT: conventional treatment, TKM: traditional Korean medicine

**Supplementary table B2. Stress level of caregivers according to the treatment experiences through complete case analysis**

|  | N | Mean | SD | F/p-value | Post-hoc |
| --- | --- | --- | --- | --- | --- |
| Stress level | | | | | |
| TKM | 35 | 6.49 | 2.24 | 7.146/≤0.001 | TKM>None  CT>None  (Dunnett T3) |
| CT | 24 | 7.42 | 2.34 |  |  |
| Both | 10 | 6.20 | 2.20 |  |  |
| None | 82 | 4.72 | 3.23 |  |  |
| Total(missing) | 151 | 5.66 | 3.01 |  |  |

CT: conventional treatment, TKM: traditional Korean medicine
